# The impact of a breast cancer educational intervention in Ghanaian high schools

**DOI:** 10.1186/s12885-022-09991-6

**Published:** 2022-08-15

**Authors:** Josephine Nsaful, Florence Dedey, Edmund Nartey, Juliana Labi, Nii Armah Adu-Aryee, Joe Nat Clegg-Lamptey

**Affiliations:** 1 Department of Surgery, University of Ghana Medical School, College of Health Sciences, University of Ghana, Accra, Ghana; 2 Department of Surgery, Korle Bu Teaching Hospital, Accra, Ghana; 3 Centre for Tropical Clinical Pharmacology & Therapeutics, University of Ghana Medical School, College of Health Sciences, University of Ghana, Accra, Ghana; 4 Department of Radiology, Korle Bu Teaching Hospital, Accra, Ghana

**Keywords:** Breast cancer, High schools, Educational intervention, Adolescents, Breast self-examination

## Abstract

**Introduction:**

Globally breast cancer is the leading cause of cancer with an estimated 2.3 million new cases and 685,000 deaths in 2020. Late presentation is the hallmark of breast cancer in Ghana for which ignorance and fear are the major reasons fuelled largely by myths and misconceptions. Breast cancer awareness and education needs to start early to bring about a change in knowledge, attitude and practices. However, Breast cancer awareness activities in Ghana have usually targeted adult women.

This study assessed the impact of breast cancer education among adolescent high school girls in Ghana.

**Methodology:**

A pre- post-test quasi experimental study was conducted at two senior high schools. A self-administered pre-educational questionnaire was followed by an educational intervention consisting of a drama, PowerPoint lecture, question and answer session and distribution of breast cancer information leaflets. After 3 months the same questionnaire was administered as a post-education test to assess the impact of the educational intervention. The total score for each domain was categorised into adequate knowledge > 50% and inadequate knowledge < 50%.

**Results:**

The number of participants in the pre-test and post-test were 1043 and 1274; the median ages [IQR] were 16.0 [15.0–17.0] for both the pre and post-test students. General knowledge on breast cancer at pre-education (29.1%) improved to 72.5% (*p* < 0.001). Knowledge on signs and symptoms improved from 33.1 to 55% (*p* < 0.001); knowledge on risk factors improved from 55.3 to 79.2% (*p* < 0.001), and knowledge on breast self-examination and screening improved from 9.8 to 22.2% (*p* < 0.001). The overall performance of the students improved from 17.2 to 59.4% (*p* < 0.001).

**Conclusion:**

There is inadequate knowledge about breast cancer and self-examination among senior high school girls in Ghana. Our breast cancer educational intervention was effective in improving general knowledge of breast cancer, risk factors, signs and symptoms and breast self-examination. The overall knowledge base improved from 17.2 to 59.4% 3 months post intervention, accompanied by an increase in the reported practice of breast self-examination and a greater belief that breast cancer is curable. This study has demonstrated the need for a school breast cancer educational program and that breast cancer education in high schools is effective.

## Introduction

Globally breast cancer is now the leading cause of cancer worldwide. Globocan estimated close to 2.3 million new cases and about 685,000 deaths from breast cancer worldwide in 2020 [1]. Out of 195 countries in the world, breast cancer had the highest cancer incidence and mortality in 159 and 110 countries respectively [2]. In Ghana breast cancer (BC) is leading in incidence with an estimated 4500 new cases and about 2000 deaths in 2020 [2]. Globocan projects approximately 3 million new cases by 2040 worldwide [3]. Despite a higher BC incidence in developed countries, a larger proportion (61%) of breast cancer deaths occur in Africa. BC occurs at a younger age in low-income countries with 50% of cases and deaths occurring in women less than 50 years. A higher portion of triple negative tumours, known to have a poorer prognosis, have also been reported [4]. The increasing breast cancer burden in Africa and other developing countries has been attributed to the increase in risk factors brought on by socioeconomic transformation. These include women having fewer children and at a later age, obesity and physical inactivity [5].

Late presentation is the hallmark of breast cancer in Ghana, with 60% of cases diagnosed at stage III and IV. Ignorance and fear, driven by myths and misconceptions are the major reasons for late presentation and non-compliance with treatment [6]. The cause of cancer has been perceived to be spiritual and several women resort to alternative treatments including herbs, prayers and homeopathy. These beliefs, attitudes and practices responsible for the late presentation, treatment and non-compliance are deeply rooted and are not unique to the uneducated patients [6, 7]. Mindsets are formed from childhood and people, when faced with a crisis, fall on their deeply-held beliefs. A call for more cancer education has been made [7].

In Ghana the past 5 years have been characterized by a surge in breast cancer awareness activities in the month of October. However, these educational campaigns are not sustained throughout the year and the target group has been women beyond school going age.

The American Cancer Society (ACS) recommends mammographic screening over clinical breast examination (CBE) and breast self-examination (BSE) as the former has been found to be more effective in reducing breast cancer mortality. However, they do acknowledge that familiarity with one’s own breast will lead to early detection of any breast changes and is of value in low resourced settings where mammography screening is not readily available [8, 9]. Promoting breast awareness including CBE and particularly the technique and practice of BSE in a setting where mammograms are few and national breast screening programs are absent can lead to early detection of breast cancer. The recommended age to start regular BSE is 20 years [8]. If myths and misconceptions are dispelled and people understand the treatment of the disease, it should in the long run translate into early presentation, compliance with treatment and a lower cancer mortality.

Research examining the knowledge and health seeking practices of the youth with regard to BC, including the impact of educational interventions among adolescents, has been undertaken in both developed [10–13] and less developed countries [14–18]. There are a few publications available from Ghana on the cancer knowledge base among the youth [19–22] but to the best of our knowledge none on the impact of breast cancer health education among adolescents. This study was designed to evaluate the BC knowledge and test the feasibility of such an intervention in Ghanaian high schools. We believe that cancer awareness and education on breast cancer needs to start at an early age to bring about a change in the knowledge, attitude and practices of our people. The aim of this study was to assess the impact of breast cancer education among high school girls in Ghana.

## Methodology

A pre- post-test quasi experimental study was conducted at two selected senior high schools, one a private co-educational school and the other a public girls’ school. Both were boarding facilities as in Ghana most senior high schools are boarding. A typical school has students who have travelled from across the regions. The headmaster/mistress takes responsibility for the students and permission was given for the school to participate in the study. A script for a short drama was given to the drama club 2 weeks ahead for them to rehearse. The procedure was first explained to the students and the option given to opt out. All consenting female students filled out a self-administered pre-educational questionnaire (pre-test). The questionnaire consisted of 21 multiple choice and true/false style questions. This covered 4 main domains on breast cancer, namely 5 questions to assess basic knowledge, 7 questions on features (signs and symptoms), 6 questions on risk factors and 3 questions on the knowledge of breast self-examination. Two additional non-domain questions were asked on respondents’ practice of breast self-examination.

Next, a short drama was performed by the drama club depicting a group of young ladies, one of whom presents with breast related symptoms. The actors are involved in a discussion which brings out some of the false information about the causes and symptoms of the disease as well as myths and misconceptions in the community. This set the stage for a 40-minute PowerPoint presentation on breast cancer. The lecture outlined what breast cancer is, risk factors, signs, symptoms, myths and misconceptions, breast self-examination and included a 1-minute video recording of the story of a breast cancer survivor. There was a question-and-answer session at the end of the presentation. Lastly, an educational leaflet on breast cancer risk, signs and symptoms and how to perform a breast self-examination was given to each student to take home and read.

After 3 months the same questionnaire was administered as a post-test to the same group of students to assess the impact of the educational intervention. The questionnaires for the pre- and post-test were not paired on the same individual, allowing students who opted out of pre-test to participate in post-test, hence our choice to use unpaired probability estimates without affecting the outcome. Each correct answer was scored 1 point and each incorrect answer or non-response a zero.

### Statistical analysis

Each of the questions in the four domains on basic knowledge of breast cancer, features of breast cancer, risk factors of breast cancer and knowledge and practise of breast self-examination was equitably scored. Each correct answer was scored one (1) point and each incorrect answer or non-response a zero. The score for each domain was calculated by summing the score of all the questions in that domain and it ranged from 0 to 5 for domain I, 0–7 for domain II, 0–6 for domain III and 0–3 for domain IV. The total score for each domain was categorised into adequate knowledge > 50% and inadequate knowledge < 50%. A Chi-square test of proportion was used to test differences in knowledge, attitude and practice of breast cancer and breast self-examination between pre-education and post-education for each question in a domain and for domain total. The overall score was calculated by summing all the scores for domain I-IV. Continuous variables were summarised as means and standard deviations (SD), and categorical variables as count and percentages. Stata 14.0® was used for the statistical analysis and *p* < 0.05 was considered statistically significant.

### Ethical considerations

Ethical approval was obtained from the Institutional Review Board of Korle Bu Teaching Hospital for Medical Research (KBTH-IRB) (study protocol ID KBTH-IRB/00063/2018). Permission was given by the heads of the schools. Written informed consent/assent was given by each participant after the procedure had been explained and the option given to withdraw at any stage. Those who opted out of the pre-test were not excluded from participating in the educational intervention.

## Results

The event was attended by 1303 school girls. The number of participants in the pre-test and post-test was 1043 and 1274 respectively giving a response rate of 80.0 and 97.8% respectively. All were females. The median ages [IQR] were 16.0 [15.0–17.0] for both the pre and post-test students.

### General knowledge on breast cancer

Table 1 shows the performance on 5 basic general knowledge questions on breast cancer (Domain I). In the pre-education test only 28.8, 18.6 and 11.4% of respondents answered correctly when asked whether breast cancer was curable, could occur in men or usually started with pain, respectively. In answer to the same questions the post-education correct answers were significantly higher (*p* < 0.001): 37.9, 81.4 and 34.7% respectively. Pre-education knowledge on women developing breast cancer below age 30 years and during pregnancy was already adequate but correct answers also improved significantly from 76.9 and 78.5% to 91.6 and 84.5% post-education respectively (*p* < 0.001).

**Table 1:** General knowledge on breast cancer

Characteristic	Correct Answer	Incorrect Answer	***p***-value
	n, (%)^a^	n, (%)^a^	
Breast cancer is curable			< 0.001
Pre-Education	300 (28.8)	743 (71.2)	
Post-Education	483 (37.9)	791 (62.1)	
Men can have breast cancer			< 0.001
Pre-Education	164 (18.6)	879 (61.2)	
Post-Education	717 (81.4)	557 (38.8)	
Women less than 30 years can have breast cancer			< 0.001
Pre-Education	802 (76.9)	241 (23.1)	
Post-Education	1167 (91.6)	107 (8.4)	
Breast cancer usually starts with pain in the breast			< 0.001
Pre-Education	119 (11.4)	924 (88.6)	
Post-Education	442 (34.7)	832 (65.3)	
Pregnant women can get breast cancer			< 0.001
Pre-Education	819 (78.5)	224 (21.5)	
Post-Education	1076 (84.5)	198 (15.5)	

^a^Row percentages

### Knowledge on signs and symptoms of breast cancer

Table 2 shows the performance on 7 questions regarding the signs and symptoms of breast cancer (Domain II). Breast cancer presenting as a lump, and nipple discharge were answered correctly in the pre-education test; 83.1, and 71.6%. These also saw significant improvement to 97.4 and 85.1% in the post-education test (*p* < 0.001). A total of 66.9% correct answers were provided for sore/rash on the breast as a sign/symptom for breast cancer in the pre-education test which improved non-significantly to 68.3% in the post-education test (*p* = 0.484). However, responses to the presence of a lump in the armpit, severe pain, change in direction of the nipple and swelling of the breast were poorly answered with correct answers in only 13.6, 4.6, 38.5 and 9.6% respectively. For these questions, although the percentage of correct answers also increased significantly (*p* < 0.001) post-education scores were still inadequate; 29.9, 9.1, 49.4 and 21.0% respectively.

**Table 2:** Knowledge on features of breast cancer

Characteristic	Correct Answer	Incorrect Answer	***p***-value
	n, %^a^	n, %^a^	
Lump in the breast			< 0.001
Pre-Education	867 (83.1)	176 (16.9)	
Post-Education	1241 (97.4)	33 (2.6)	
Lump in the armpit			< 0.001
Pre-Education	142 (13.6)	901 (86.4)	
Post-Education	381 (29.9)	893 (70.1)	
Sore/rash on the breast			0.484
Pre-Education	698 (66.9)	345 (33.1)	
Post-Education	870 (68.3)	404 (31.7)	
Severe breast pain			< 0.001
Pre-Education	48 (4.6)	995 (95.4)	
Post-Education	116 (9.1)	1158 (90.9)	
Change in direction of the nipple			< 0.001
Pre-Education	401 (38.5)	642 (61.5)	
Post-Education	629 (49.4)	645 (50.6)	
Fluid discharge from the nipple			< 0.001
Pre-Education	747 (71.6)	296 (28.4)	
Post-Education	1084 (85.1)	190 (14.9)	
Swelling or increase in size of breast			< 0.001
Pre-Education	100 (9.6)	943 (90.4)	
Post-Education	267 (21.0)	1007 (79.0)	

^a^Row percentages

### Knowledge on risk factors for developing breast cancer

Knowledge on risk factors for breast cancer is represented in Table 3 (Domain III). With the exception of the question on parity less than 50% correctly identified family history, breastfeeding, alcohol and lack of exercise as risk factors in the pre-education test. Knowledge of these risk factors did increase significantly post-education (*p* < 0.05). As many as 94% consistently inaccurately believe handkerchiefs/mobile phones placed in a brasier is a risk for developing breast cancer both before and after the intervention.

**Table 3:** Knowledge on factors increasing risk of getting breast cancer

Characteristic	Correct Answer	Incorrect Answer	***p***-value
	n, %^a^	n, %^a^	
Family member had breast cancer			< 0.001
Pre-Education	343 (32.9)	700 (67.1)	
Post-Education	752 (59.0)	522 (41.0)	
Breastfeeding			< 0.001
Pre-Education	474 (45.5)	569 (54.5)	
Post-Education	925 (72.6)	349 (27.4)	
Having more than 5 children			< 0.001
Pre-Education	824 (79.0)	219 (21.0)	
Post-Education	1080 (84.8)	194 (15.2)	
Keeping handkerchief/mobile phone in your brasier			0.693
Pre-Education	59 (5.7)	984 (94.3)	
Post-Education	77 (6.0)	1197 (94.0)	
Excessive alcohol intake			< 0.001
Pre-Education	498 (47.7)	545 (52.3)	
Post-Education	781 (61.3)	493 (38.7)	
Lack of exercise			0.020
Pre-Education	453 (43.4)	590 (56.6)	
Post-Education	615 (48.3)	659 (51.7)	

^a^Row percentages

### Knowledge on breast self-examination and breast cancer screening

Table 4 summarizes the knowledge on breast self-examination (BSE) and breast screening (domain IV). Knowledge on the frequency and timing of performing a BSE was correctly answered by 29.2 and 42.3% of respondents pre-education and 43.3 and 45.9% post-education respectively which was significant (*p* < 0.001). The question on the recommended age at which women are to start screening mammograms was very poorly answered both pre- and post-education, with only 2.6 and 5.3% answering correctly (*p* = 0.080).

**Table 4:** Knowledge on breast self-examination and screening

Characteristic	Correct Answer	Incorrect Answer	***p***-value
	n, %^a^	n, %^a^	
Frequency of doing breast Self-examination			< 0.001
Pre-Education	305 (29.2)	738 (70.8)	
Post-Education	551 (43.3)	723 (56.7)	
Best time to do breast self-examination			0.080
Pre-Education	441 (42.3)	602 (57.7)	
Post-Education	585 (45.9)	689 (54.1)	
Age at which a woman should have first screening mammogram in Ghana			< 0.001
Pre-Education	27 (2.6)	1016 (97.4)	
Post-Education	68 (5.3)	1206 (94.7)	

^a^Row percentages

Pre-education, 79.3% (*n* = 827) of students had heard of breast self-examination. Sub-group analysis indicated that of these, 38.6% (*n* = 319) had practiced breast self-examination. This proportion of students who did breast self-examination significantly increased (*p* = 0.005) to 44.9% (*n* = 514) among the 1146 post-education students who had heard of breast self-examination (data not shown).

### Overall performance on breast cancer knowledge

Table 5 represents the analysis of each of the 4 domains assessed and an overall assessment of the knowledge pre- and post-education. Adequate knowledge is categorised as > 50% correct answers and inadequate knowledge as < 50% correct answers. The overall general knowledge on breast cancer (domain I) at pre-education was rather inadequate at 29.1% and improved significantly to 72.5% (now adequate) post-education (*p* < 0.001). The knowledge on features (signs and symptoms) of breast cancer (domain II) also started off as inadequate (33.1%) pre-education and improved significantly to a low adequate score of 55% (*p* < 0.001). The assessment of knowledge on risk factors (domain III) pre-education was just adequate with 55.3% correct answers, but post-education saw a significant improvement to 79.2% (*p* < 0.001). The overall knowledge on BSE and screening (domain IV) saw a significant improvement from 9.8 to 22.2% (*p* < 0.001) but remained inadequate. Finally, the overall performance of the students improved significantly from pre-education 17.2% to post-education 59.4% (*p* < 0.001).

**Table 5:** Domain analysis

Characteristic	Adequate	Inadequate	***p***-value
	n, %^a^	n, %^a^	
Domain I			
General knowledge on breast cancer			< 0.001
Pre-Education	304 (29.1)	739 (70.9)	
Post-Education	924 (72.5)	350 (27.5)	
Domain II			
Knowledge on features of breast cancer			< 0.001
Pre-Education	345 (33.1)	698 (66.9)	
Post-Education	700 (55.0)	574 (45.0)	
Domain III			
Knowledge on factors increasing risk of getting breast cancer			< 0.001
Pre-Education	577 (55.3)	466 (44.7)	
Post-Education	1009 (79.2)	265 (20.8)	
Domain IV			
Knowledge on breast self-examination and screening			< 0.001
Pre-Education	102 (9.8)	941 (90.2)	
Post-Education	283 (22.2)	991 (77.8)	
Overall			
Overall knowledge			< 0.001
Pre-Education	179 (17.2)	864 (82.8)	
Post-Education	757 (59.4)	517 (40.6)	

^a^Row percentages

## Discussion

This study has demonstrated that the designed educational intervention (drama, lecture, question-and-answer session, and educational leaflets) significantly improved knowledge in all domains on breast cancer risk factors, signs and symptoms, breast self-examination and the practice of BSE significantly among adolescent females. The overall knowledge on breast cancer improved after the intervention from 17.2 to 59.4% (*p* < 0.001). The greatest improvement was seen in general knowledge about breast cancer from 29.1 to 72.5% (*p* < 0.001) and the least improvement in knowledge on BSE from 9.8 to 22.2% (*p* < 0.001).

It has been established that the level of knowledge about breast cancer among the youth is inadequate. A close look at the domains pre-education reveals very low scores for general knowledge (29.1%) and features of breast cancer (33.2%), and even lower scores for breast self-examination 9.8%. This is similar to breast cancer knowledge levels in other low and middle income countries (LMIC) such as Nigeria [16, 23], India [24], Bangladesh [15] and Siri Lanka [18]. This phenomenon is however, not restricted to LMIC. Adolescents in developed countries have also been found to be deficient in breast cancer knowledge. College and high school students in the USA have been found to have poor knowledge on breast cancer, its risk factors and BSE, scoring a mean of 13.5 ± 0.33 (high school students) and 15.5 ± 0.32 (college students) out of a total score of 30 [10]. This inadequate breast cancer knowledge among the youth worldwide makes a case for the introduction of breast cancer education targeted at adolescents.

This study was innovative in using a multi-tooled approach: first a drama acted out by the students, then a PowerPoint lecture given by doctors, followed by a question-and-answer session and finally breast cancer information leaflets for participants to take home and read. Others have applied various educational methods, all with good results. An hour long lesson has been proven to improve the knowledge base of girls on breast cancer and BSE [25]. A quasi-experimental research carried out among Nigerian adolescents used BSE pamphlets, and testing done 8 weeks later found an increase in BSE knowledge and perception [26]. Another publication from Nigeria demonstrated that a 45–60 minute educational session utilizing PowerPoint, video and demonstration of BSE resulted in a significant improvement in knowledge, attitude and the practice of BSE 8 weeks after intervention [27]. This study utilized students to act out a drama in order to make learning a collaborative experience. Also in Nigeria, a study found peer education to be an effective tool and a cost effective means of breast cancer education among adolescents [16].

The use of peer educators has demonstrated similar success in Egypt [28]. A study carried out among adolescents in Mexican middle schools utilized an educational intervention which included a reading guide that was later discussed at a plenary session. Likewise, this saw significant learning with 53% correct answers pre-intervention increasing to 75% correct answers post-intervention [29]. In Saudi Arabia an all-female team of doctors visiting schools employed a series of short lectures, discussion groups and role playing on the technique of breast examination. They found that not only did the mean knowledge indexes for breast cancer and BSE improve after an educational session, but also some girls (27%) had started practicing BSE over the 6-month period post intervention [30].

In a comparison of breast health teaching methods, it was found that interactive teaching methods with simulated breast models resulted in higher knowledge retention 4 weeks after the intervention compared to the traditional didactic teaching method. It is noteworthy however, that there was still significant improvement in knowledge in both the traditional didactic and the interactive methods [12]. Indeed, the use of demonstration methods and audio-visual media has been found to be a successful means of breast cancer and BSE education among adolescents [14]. Recently the impact of social media has been explored and the use of youth-targeted YouTube-styled videos has been promising in educating adolescents on the breast cancer risk associated with smoking [11]. These studies all prove that various teaching tools and if possible as was utilized in our study, a combination of teaching methods is effective in achieving learning in breast cancer education.

For breast cancer education to be effective there should be a translation of the knowledge into appropriate health seeking and preventive practices and the appropriate attitude should one detect any breast changes or be diagnosed with breast cancer. For instance, a study done in a Ghanaian University of Allied Health found that at least 70% were aware of breast cancer, mammography and BSE, but this knowledge did not influence their behaviour, as only 43% practiced BSE and 46% of these students felt there was no chance that they might develop breast cancer in the future, while 16% were uncertain of their risk. Not surprisingly, those who felt there was no risk or did not know their risk were less likely to perform BSE than those who perceived some risk [31]. Though the level of breast cancer awareness and BSE awareness in Malaysia was as good as 87.6 and 60.6% respectively, the knowledge on BSE was poor (40.4%) [32]. In this study we found that our educational intervention did not change the perception (in 94% of participants) that handkerchiefs/mobile phones placed in a brasier is not a risk for developing breast cancer (*p* = 0.693). The knowledge that sore/rash is a symptom of breast cancer also did not significantly improve from 66.9 to 68.3% (*p* = 0.484). Knowledge on the recommended age at which women are to start screening mammograms also showed minimal improvement from 2.6 to 5.3% (*p* < 0.001). This phenomenon is not unique to our study. For instance, the impression that pain and weight loss were not the first symptoms of breast cancer was not corrected after the Nigerian educational intervention [16].

On the other hand, our intervention did see a significant increase in the number of students practicing BSE 3 months post-intervention from 38.6 to 44.9% (*p* = 0.005). Notably, more girls now believed that BC is a curable disease, from 28.8% pre-intervention to 37.9% post-intervention (*p* < 0.001). Likewise, an educational intervention in the UK was found to have a sustained improvement in breast knowledge and attitudes 3 and 6 months later [13]. A quasi-experimental study in Korea found that a breast cancer educational intervention did improve all aspects of learning a week after the intervention. However, 3 months later breast cancer knowledge and attitude on prevention were sustained but the improvement was not sustained for self-efficacy and behavioural intentions. This was attributed to the ability of short-term interventions to change one’s knowledge but not necessarily the social cognitive factors that would reflect in a sustained behaviour change. Such sustained behaviour changes would take long-term interventions involving several booster sessions [33].

An innovative educational intervention carried out in Mexico went further to determine the impact on the female relatives of the participants and it was found that there was transference of knowledge so that breast cancer knowledge of relatives at home saw improvement from 55 to 61% 4 months post-intervention. This demonstrates a potential strategy for public education and change in societal norms [34]. Breast cancer educational programs should be designed to achieve sustained gains in knowledge and long-term behavioural change in the community.

Social media, teachers and electronic media have been found to be the leading sources of information on breast cancer and little from health professionals [31, 32]. Getting already limited and constrained healthcare professionals into every school and every classroom, though desirable, may not be practical. There is a need for innovation in getting cancer education into schools either as part of the curriculum or seasonal school activities. Schoolgirls themselves have concerns about and admit the need for breast cancer education [35]. A case for schools to lead in cancer health education has been made in a commentary by Morse in which he makes reference to the guidelines and scope of such a program drawn up as far back as 1995 by the ACS [36]. The Centre for Disease Control (CDC) also recommends that the youth be taught cancer protective behaviours which include good nutrition, physical activity, human papilloma virus (HPV) vaccination and reducing harmful exposures to smoking, alcohol, tanning, certain chemicals, etc [37].

In Portugal a training program for biology teachers was found to be an effective tool in increasing teachers’ knowledge and perception of cancer and resulted in an increase also in students’ knowledge on cancer. This project covered education in breast, cervical, colorectal and skin cancer [38]. A Cancer Education Partnership Program has been developed in the USA for underserved schools. This introduced children as young as third-grade through to high school to the concept of cancer, exposing them to basic knowledge about cancer risks, prevention, nutrition and more advanced oncology, genetics and biotechnology as they got older [39]. 2020 saw the introduction of health education in UK curricula which includes preventive lifestyles and awareness of cancer screening [40].

Schools present the perfect opportunity to gain access to a nation’s youth and it should be possible to introduce breast cancer education in schools in Ghana and other LMICs. It would be prudent to train teachers to be the primary source of information. A collaboration between the Health and Education Sectors would be beneficial in organizing training programs and developing materials for the curriculum/syllabus. This need not be restricted to breast cancer but can be extended to other cancers and diseases of public health importance. The schools’ efforts could then be augmented by a team of health professionals from the school locality. These healthcare workers could be given access to schools periodically during awareness months to have a more detailed interaction with students. Our experience on school visits has been characterized by enthusiasm and cooperation both on the side of the school authorities/teachers and the students. We believe that such programs will be a welcome introduction in our schools.

## Conclusion

This paper set out to assess the impact of breast cancer education among high school girls in Ghana and we observed that there is inadequate knowledge about breast cancer and breast self-examination among senior high school girls in Ghana. This breast cancer educational intervention effectively improved general knowledge in breast cancer, knowledge on risk factors, signs and symptoms and breast self-examination. The overall knowledge base improved (by 42.2%) 3 months post intervention. This was accompanied by a significant increase in the practice of breast self-examination and in the understanding that breast cancer is a curable disease. This study has demonstrated the need for a school breast cancer educational program and proven that breast cancer education in high schools is effective.

We recommend that breast cancer education should be introduced into the school curricula, supported by local healthcare professionals. Results of this study indicate that, other than the fundamental facts about the disease, areas that need emphasis should include addressing the myths and misconceptions and teaching the practice of BSE. For breast cancer education to achieve the desired sustained, behaviour changing effect in LMIC we recommend the use of a multi-tooled, culturally acceptable, cost-effective educational approach involving multiple sessions throughout the school year.

This study was limited in that it did not pair the pre and post-test questionnaires, but its strength lies in the large sample size. The study has developed an educational tool for use by health professionals which can be used at school visits for the purpose of breast cancer education. This study has laid the foundation for further research to be done in adolescent cancer education in Ghana and for the development of educational tools in other cancers.

## Data Availability

The data and materials of this study are freely available at 10.6084/m9.figshare.20021963

## Abbreviations

BC: Breast Cancer
BSE: Breast Self-Examination
CBE: Clinical Breast Examination
LMICs: Low and Middle Income Countries
ACS: American Cancer Society
CDC: Centre for Disease Control
